# Editorial: Physical activity: a promising modifiable behavior to protect brain, cognition, and mental health across the lifespan

**DOI:** 10.3389/fpsyg.2025.1636754

**Published:** 2025-07-02

**Authors:** Jose Mora-Gonzalez, Yaira Barranco-Ruiz, Darío Bellón, María Rodriguez-Ayllon

**Affiliations:** ^1^Department of Physical Education and Sports, Faculty of Sport Sciences, Sport and Health University Research Institute (iMUDS), University of Granada, Granada, Spain; ^2^Biomedical Research Institute of Málaga and Nanomedicine Platform (IBIMA Plataforma BIONAND), Málaga, Spain; ^3^Prevention and Health Promotion Research Network (redIAPP) and Chronicity, Primary Care and Health Promotion Research Network (RICAPPS), Instituto de Salud Carlos III (ISCIII), Madrid, Spain; ^4^Department of Epidemiology, Erasmus University Medical Center, Rotterdam, Netherlands

**Keywords:** exercise, mental health, sleep, healthy habits, brain health, cognition, prevention

## 1 Introduction

Physical activity is increasingly recognized as a pivotal modifiable behavior with significant impacts on physical health, brain function, cognition, and mental wellbeing throughout life (Katzmarzyk et al., 2022; Domingos et al., 2021; Zhang et al., 2023; Erickson et al., 2019). Despite overwhelming evidence supporting its positive effects, global physical activity levels remain alarmingly low, presenting a significant public health challenge (Strain et al., 2024; World Health Organization, 2024; Guthold et al., 2020; Bull et al., 2020). This Research Topic highlights the powerful role of physical activity in promoting brain health and mental wellbeing, focusing on cognitive function, emotional regulation, and psychological distress across diverse populations and life stages. It also provides insights into some mechanisms linking physical activity with brain health outcomes.

## 2 Overview of contributions

### 2.1 Physical activity's role in youth: cognition, mental health, and development

Cui et al. explored the relationship between physical activity and cognitive abilities in Chinese adolescents. Their findings suggested that higher levels of physical activity are linked to better cognitive performance, especially in adolescents who initially showed lower cognitive abilities. The study also highlighted self-education expectations and learning behaviors as potential mechanisms linking physical activity with cognitive performance.

Building on this, Yan et al. examined how physical activity relates to mental health in middle school students from China. Their study emphasized that physical activity can enhance psychological wellbeing by reducing negative emotions and boosting self-efficacy. These two variables appear to be important pathways through which physical activity supports overall mental health during adolescence.

Zhang et al. conducted a systematic review and meta-analysis of 13 studies to examine brain activation patterns in children and adolescents engaged in physical activity. The analysis revealed consistent activation in frontal associative brain regions, such as the medial, middle, and inferior frontal gyri. Notably, exercises with greater variety elicited more activation in these areas, emphasizing the importance of incorporating diverse activities into intervention practices. The findings suggest that promoting variability in physical activities can optimize brain function in young individuals.

Complementing these studies, Xiang et al. explored how physical activity is related to academic achievement in Chinese secondary school students. They identified three pathways linking physical activity to better academic outcomes: social support, learning engagement, and a combination of both, where physical activity boosts social support, enhancing learning engagement and academic success. These findings suggest that physical activity can benefit students academically by fostering stronger social connections and greater engagement in learning.

### 2.2 Physical activity, self-perception, and mental health in college students

Jankauskiene and Baceviciene's study examined how mindfulness during physical activity relates to body appreciation among Lithuanian college students, considering the roles of sex and BMI. They found that mindfulness is linked to greater body appreciation, especially in individuals with higher BMI, highlighting the importance of integrating practices like present-moment awareness and body acceptance into physical activity.

Ke et al. studied the connection between physical activity and smartphone addiction in Chinese college students, showing that self-esteem mediates this relationship. Overall, regular physical activity was associated with lower smartphone dependency, suggesting that improving self-esteem through exercise could help manage technology-related behaviors.

Gutiérrez-Capote et al. explored the acute effects of physical activity on cognitive performance through basketball drills among Spanish college students. Their findings showed that even short bouts of physical activity can enhance cognitive function, suggesting benefits for educational settings where brief activity breaks may improve focus and learning.

### 2.3 Physical activity, depression, cognition, and trauma recovery in adults

Ibáñez et al. studied the relationship between physical activity, sedentary behavior, and depressive symptoms in Spanish adults with major depressive disorder (MDD) during different stages of the COVID-19 pandemic. They found that more sedentary time correlated with higher depressive symptoms, while light physical activity was linked to lower symptoms, except during strict lockdowns. This underscores the importance of reducing sedentary behavior and encouraging physical activity to manage depression, particularly during public health fluctuations.

Sewell et al. studied whether sleep mediates the effects of exercise on cognition in older adults in Australia. Acute high-intensity exercise showed no immediate effect on cognition or sleep, nor did sleep mediate this relationship. However, changes in light and deep sleep from baseline to post-intervention were linked to improvements in episodic memory at ~24 h, regardless of the intervention. These findings suggest that while exercise did not directly affect cognition or sleep, sleep quality may independently enhance cognitive performance, highlighting the need for further research into the relationship between sleep, exercise, and cognition in this specific population.

Lastly, SantaBarbara et al. explored the associations of exercise with post-traumatic stress disorder (PTSD) symptoms, distress, pain, and sleep in trauma-exposed adults. Overall, participants meeting exercise guidelines reported reduced PTSD symptoms, lower distress and pain, and better sleep quality. The findings support exercise as a valuable therapeutic tool for improving mental health and resilience in trauma survivors.

## 3 Insights and future directions, and general conclusions

The studies in this Research Topic highlight the benefits of physical activity for brain, cognitive function, emotional regulation, and mental health across the lifespan. However, several areas warrant further exploration. Future research should focus on personalized physical activity interventions that consider individual preferences and characteristics, such as age, gender, and health status. For protecting mental health, physical activity types should align with personal preferences—whether team sports, yoga, or mindful movement practices—rather than focusing solely on aesthetic and general benefits. Promoting self-awareness during physical activity, such as mindfulness or body awareness, could further enhance engagement and adherence.

Notably, exercises with greater variety elicited more activation in key brain areas, emphasizing the importance of incorporating diverse activities into intervention practices. Promoting variability in physical activities can optimize brain function in young individuals. On the other hand, when used mindfully, smartphones can promote physical activity and support healthy lifestyle changes among youth, such as increasing physical activity. Instead of demonizing technology as a driver of sedentary behavior, efforts should focus on encouraging its responsible use. Enhancing digital literacy is key to helping young people harness technology's benefits while maintaining a balanced lifestyle.

Moreover, more long-term randomized controlled trials are needed to confirm the findings presented in this Research Topic, which primarily includes observational and short-term intervention studies. Finally, environmental factors, such as social contexts and external restrictions—like pandemic-related lockdowns, access to exercise facilities, or cultural barriers—can significantly impact the effectiveness of exercise interventions. Future research should consider these variables to ensure broad participation and maximize the benefits.
